# Anaphylaxis to cow's milk protein in a probiotic not detected by the electronic medical record

**DOI:** 10.1002/jpr3.12128

**Published:** 2024-09-11

**Authors:** Jonathan E. Teitelbaum, Joseph Dallessio, Jacqueline Brunetto, Jacqueline A. Ross

**Affiliations:** ^1^ Department of Pediatrics The Unterberg Children's Hospital at Monmouth Medical Center Long Branch New Jersey USA; ^2^ Allergy Partners of NJ Ocean New Jersey USA

**Keywords:** excipient, food allergy, supplement

## Abstract

A 13‐year‐old female with a history of congenital left lower leg lymphedema, multiple food allergies, including an immunoglobulin E mediated severe cow's milk allergy, and well‐controlled moderate persistent asthma was hospitalized with left lower leg erysipelas and Group A Streptococcus septicemia. While hospitalized, immediately after exposure to cow's milk protein as an inactive ingredient within a probiotic, she developed anaphylaxis with respiratory failure requiring intubation. This is only the third reported case of anaphylaxis due to a probiotic. Additionally, it raises issues inherent to the electronic medical record with respect to its inability to identify allergens in supplements as opposed to medications.

## 1 INTRODUCTION

Probiotics are “good bacteria” that confer a health benefit on the host. While there is a focus on medication safety, probiotic safety garners less attention. A 2009 review of probiotic safety^1^ concluded there was no significant increase in risk associated with probiotic use. A 2014 systematic review identified the main potential adverse effects of probiotics as sepsis, fungemia, and gastrointestinal ischemia. However, Tidari concluded the overwhelming evidence indicates that probiotics are safe.^2^ A recent paper^3^ makes recommendations regarding probiotics including acute safety, long‐term safety, and general recommendations (e.g., probiotic quality, adverse event reporting, product labeling).

Few have focused on safety concerns related to the inactive ingredients within probiotics. Cow's milk protein is a frequent trigger of allergic reactions, including anaphylaxis. A 2019 review of allergic reactions to cow's milk proteins in medications^4^ revealed lactose as an excipient, contaminated by cow's milk protein, in dry‐powder inhalers and intravenous methylprednisolone sodium succinate has caused allergic reactions.

We describe an anaphylactic reaction to a probiotic in an adolescent with cow's milk protein allergy.

## 2 CASE REPORT

A 13‐year‐old female with congenital left lower extremity lymphedema presented with fever, progressive left lower leg erythema, and swelling. The blood culture grew Group A Streptococcus (pyogenes) and she was treated with ampicillin.

She had well‐controlled moderate persistent asthma and multiple immunoglobulin E mediated food allergies to various proteins, including cow's milk, egg, peanut, tree nuts, and seeds. She had a history of anaphylaxis to cow's milk protein. Her last skin testing in 2022 documented 4+ reactions to cow's milk and egg.

On hospital day three she was afebrile, with normal vital signs. Secondary to loose stools, she requested a probiotic for antibiotic‐associated diarrhea. Previously she had taken a probiotic (*Lactobacillus rhamnosus GG*) at home without difficulty. She was given four tablets of Lactobacillus (Brookfield Pharmaceuticals [*Lactobacillus acidophilus, Lactobacillus bulgaricus*]). Within 15 min, she complained of a scratchy throat and difficulty breathing. Her heart rate was 150 beats per minute, respiratory rate was 29 breaths per minute, blood pressure was 75/44 mm of mercury (mmHg), and oxygen saturation was below 60%. She had mental status change, cyanosis, urticaria, and bilateral poor air entry. She was diagnosed with anaphylaxis secondary to the probiotic. Two doses of epinephrine 0.3 mg were given intramuscularly. Supplemental oxygen via a nonrebreather mask was started. She was electively intubated using propofol and midazolam. The airway was edematous with a Cormack‐Lehane Classification grade IV. Poor aeration with wheezing warranted a third epinephrine dose. Nebulized albuterol, intravenous magnesium sulfate, methylprednisolone, and ketamine were administered. The arterial blood gas revealed a pH of 6.89, partial pressure of oxygen (PaO_2_) of 67 mmHg and a partial pressure of carbon dioxide (PaCO_2_) of 107 mmHg. Steroids were continued and continuous albuterol therapy via ventilator, a magnesium sulfate drip, and famotidine were started. Respiratory acidosis improved but acute respiratory distress syndrome (ARDS) ensued. Twenty hours later, she developed biphasic anaphylaxis with urticaria, bronchospasm, and oxygen desaturation. She received two more doses of epinephrine.

The probiotic inactive ingredients included: sugar, whey, microcrystalline, cellulose, stearic acid, lactose monohydrate, croscarmellose sodium, maltodextrin, nonfat milk, magnesium stearate, and silicon dioxide. In this institution, supplements such as probiotics are repackaged at a central location, removed from the original container, and placed into individual packages without information from the label indicating inactive ingredients. The electronic medical record (EMR) does not cross‐reference allergy information for supplements or their inactive ingredients with the documented patient allergies.

All symptoms improved. She remained mechanically ventilated for 6 days. Eleven days after admission she was discharged to home.

## 3 DISCUSSION

Anaphylaxis to a probiotic is rare, with two prior case reports in the literature. The first, an infant with cow's milk protein allergy who developed urticaria and laryngeal edema 15 min after a probiotic.^5^ These authors describe skin prick testing of probiotic products in children with milk allergy.^6^ The first, second and third tested probiotic products elicited a positive skin test in 100%, 70%, and 0% of the children, respectively. Immunoblot with polyclonal antibeta‐lactoglobulin IgG confirmed the presence of beta‐lactoglobulin in the probiotic products.

The second case report was a 6‐year‐old with multiple food protein allergies including cow's milk, eggs, fish, kiwi, and hazelnut who developed mouth and pharyngeal pruritus, erythema, edema, vomiting, and dizziness immediately after sipping a probiotic solution.^7^ Skin prick test to this probiotic was positive. Enzyme linked immunosorbent assay (ELIZA) of the probiotic detected cow's milk and egg white proteins. Using ELISA, they found that 10 of 11 tested probiotic products had cow's milk protein and three had egg protein. Of those with milk, only two warned that it contained milk, four warned of lactose, one of lactic acid, one stated it was free of cow's milk, and two did not provide information on cow's milk content. None warned of egg content.

A study evaluating oral dosage formulations of the most frequently prescribed medications revealed that they consist of 75% inactive ingredients.^8^ Accordingly, 41% of all drug products contain more than 250 mg of inactive ingredients with the average tablet or capsule containing 8.8 inactive ingredients. A total of 38 inactive ingredients have been reported to cause allergic symptoms. The authors emphasize the need for more detailed datasets defining these components to assist pharmacists, clinicians and patients.

Since inactive ingredients can include antigenic food proteins, careful review is needed before administration to food allergic individuals.^9^ Our patient had a documented milk allergy in the EMR. EMRs recognize potential allergic cross reactivity among medications by utilizing a variety of databases.^10^ However, First Databank, the program used in our facility, does not include supplements. Thus, cow's milk within the probiotic, did not trigger an allergy alert for the clinician or pharmacist to prevent dispensing of the product to our patient with a documented food allergy. This illustrates an opportunity to improve systems within the hospital to identify potential allergens in supplements and prevent future allergic reactions. Solutions include establishing and incorporating database information about supplements and their inactive ingredients into the EMR. Some institutions have elected not to provide supplements in the hospital setting. Alternatively, pharmacists can select supplements that do not contain allergens. Finally, the pharmacist can perform a manual allergy cross‐check when a supplement is ordered.

## CONFLICT OF INTEREST STATEMENT

The authors declare no conflict of interest.

## ETHICS STATEMENT

Verbal witnessed consent was obtained from the patient's guardians for publication of the case details as the patient is underage.
